# Supplementary material for: AdapTor: Adaptive Topological Regression for quantitative structure–activity relationship modeling
Source: J Cheminform. 2025 Aug 28;17:128. doi: 10.1186/s13321-025-01071-8 (PMC12392520; doi:10.1186/s13321-025-01071-8)
Supplement: Supplementary file 1 — Supplementary Material 1. [file 13321_2025_1071_MOESM1_ESM.pdf]

Supplementary Material for "AdapToR: Adaptive Topological Regression for quantitative structure-activity relationship modeling"

Table S1: Number of drugs for 60 cell lines in the NCI60 dataset

Cell Line	# of drug[1]	Cell Line	# of drug[1]	Cell Line	# of drug[1]
786-O	48701	K562	48264	RXF 393	44464
A498	43186	LOX IMVI	46786	SF268	49153
A549	49910	M14	48659	SF295	49356
ACHN	49002	MALME-3M	45808	SF539	46680
BT549	33183	MCF7	36618	SK-MEL-2	46433
CAKI	46706	MDA-MB-231	36009	SK-MEL-28	48801
CCRF-CEM	46820	MDA-MB-435	36573	SK-MEL-5	48560
Colo205	49065	MOLT-4	48608	SK-OV-3	47124
DU145	36321	NCI-ADR-RES	36855	SN12C	49218
EKVX	46990	NCI-H226	45981	SNB-19	49017
HCC2998	44474	NCI-H23	49310	SNB-75	45957
HCT-116	49184	NCI-H322M	48066	SR	40869
HCT-15	49219	NCI-H460	48230	SW620	49784
HL-60 (TB)	44764	NCI-H522	45347	TK-10	48137
HOP-62	48208	OVCAR-3	48270	T-47D	34468
HOP-92	43665	OVCAR-4	47199	U251	49466
HS 578T	34512	OVCAR-5	48478	UACC-257	48992
HT29	49145	OVCAR-8	49794	UACC-62	48549
IGROV1	48925	PC-3	36407	UO-31	48917
KM12	49282	RPMI-8226	46236	**MDA-MB-468**[2]	5566

[1] Drugs with invalid SMILES are excluded.
[2] Cell line MDA-MB-468 was excluded from the analysis due to a limited number of available drugs.

Table S2: Model performance averaged over 530 ChEMBL datasets and 5-fold cross-validation splits

Model	NRMSE	Spearman	Train time (sec)	Test time (sec)
TCNN	0.660	0.744	30.6	6.9
TCNN-Aug	0.596	0.786	109.8	20.0
TR	0.626	0.763	**1.6**	1.0
Ensemble TR[1]	0.599	0.785	13.8	12.3
AdapToR	**0.586**	**0.791**	**1.7**	**0.2**

[1] Ensemble TR in this table refers to Ensemble TR described in [2], which does not incorporate any new model features proposed in the main text.

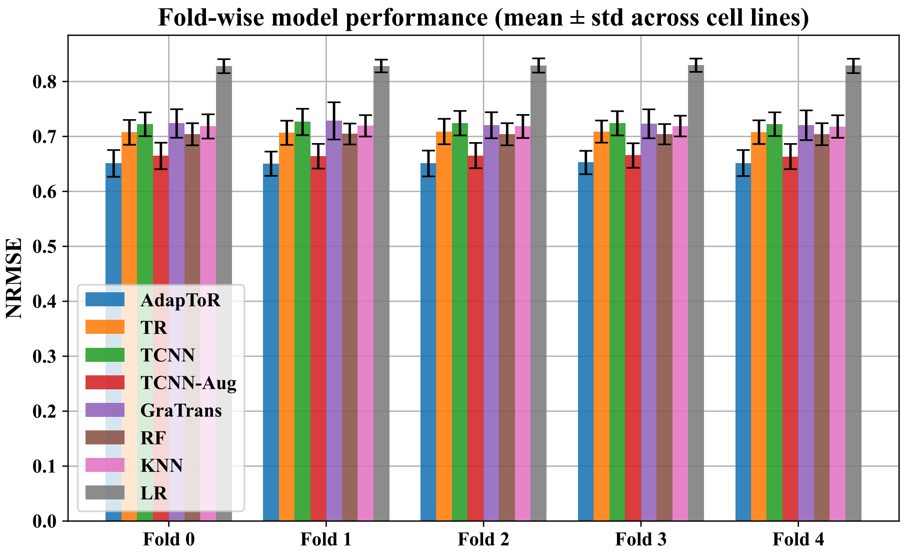

Figure S1: Bar plot showing fold-wise model performance in terms of NRMSE. Each bar represents the mean NRMSE across all cell lines for a given cross-validation fold, and the error bars indicate the corresponding standard deviation.

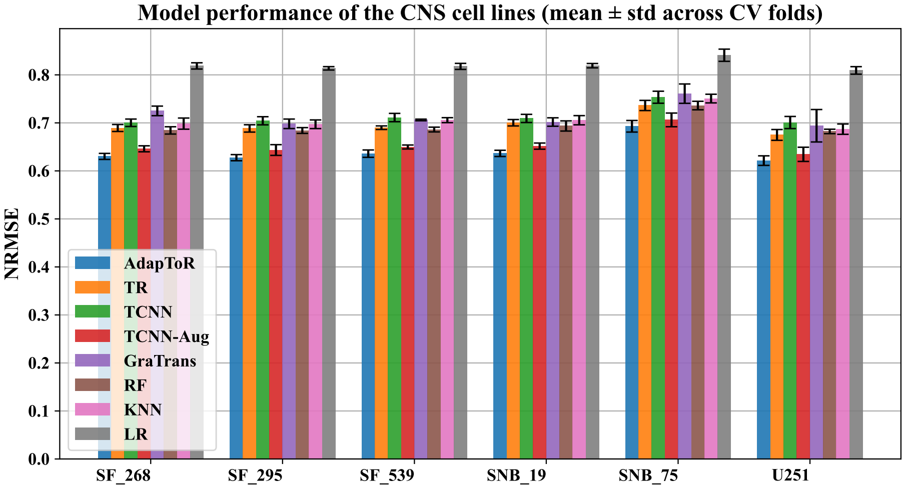

Figure S2: Bar plot showing cell line-wise model performance in terms of NRMSE for the cell lines corresponding to the central nervous system (CNS) cancer. Each bar represents the mean NRMSE across 5 cross-validation folds for a given cell line, and the error bars indicate the corresponding standard deviation.

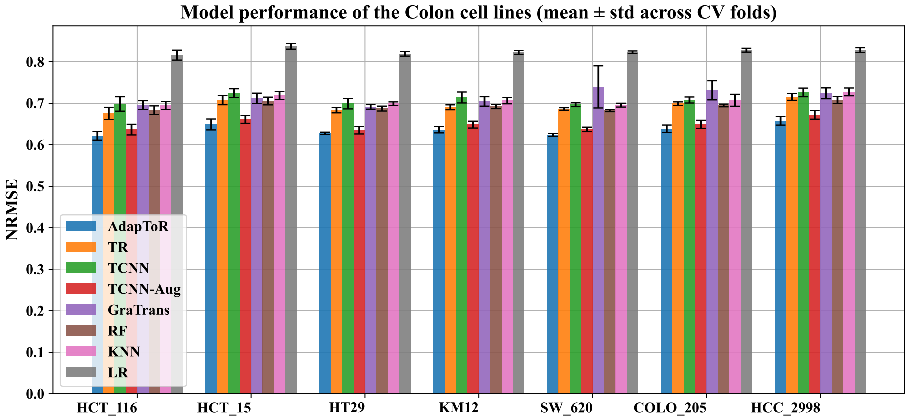

Figure S3: Bar plot showing cell line-wise model performance in terms of NRMSE for the cell lines corresponding to the colon cancer. Each bar represents the mean NRMSE across 5 cross-validation folds for a given cell line, and the error bars indicate the corresponding standard deviation.

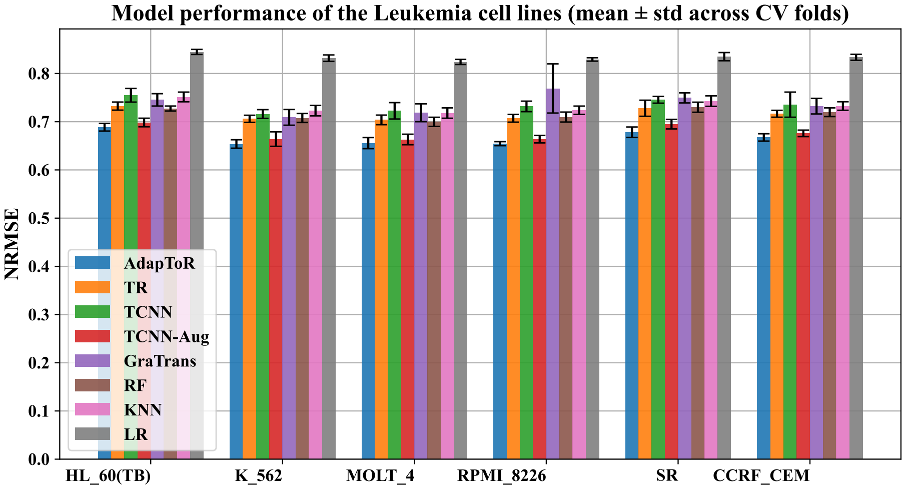

Figure S4: Bar plot showing cell line-wise model performance in terms of NRMSE for the cell lines corresponding to the leukemia cancer. Each bar represents the mean NRMSE across 5 cross-validation folds for a given cell line, and the error bars indicate the corresponding standard deviation.

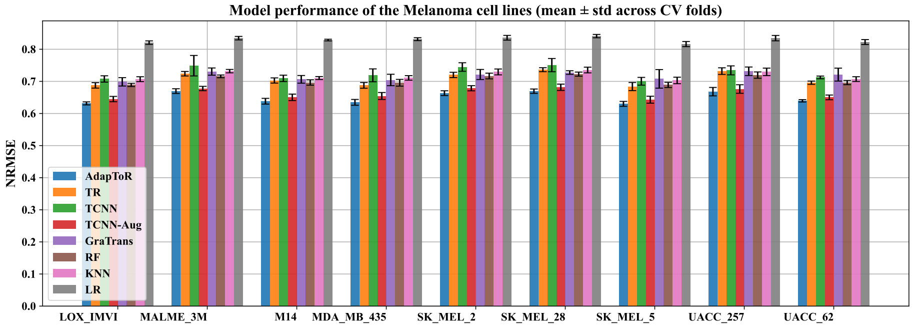

Figure S5: Bar plot showing cell line-wise model performance in terms of NRMSE for the cell lines corresponding to the melanoma cancer. Each bar represents the mean NRMSE across 5 cross-validation folds for a given cell line, and the error bars indicate the corresponding standard deviation.

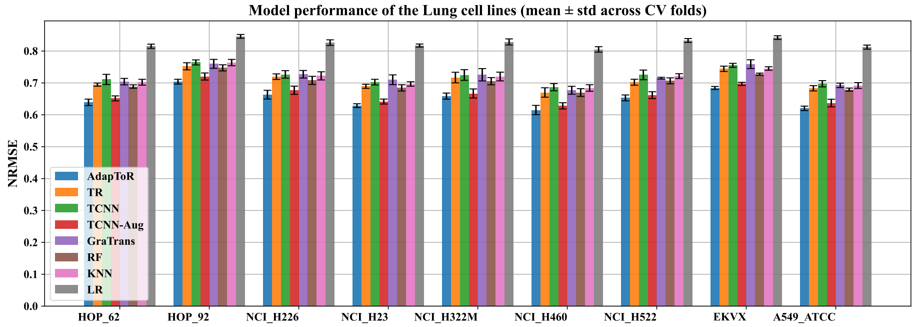

Figure S6: Bar plot showing cell line-wise model performance in terms of NRMSE for the cell lines corresponding to the lung cancer. Each bar represents the mean NRMSE across 5 cross-validation folds for a given cell line, and the error bars indicate the corresponding standard deviation.

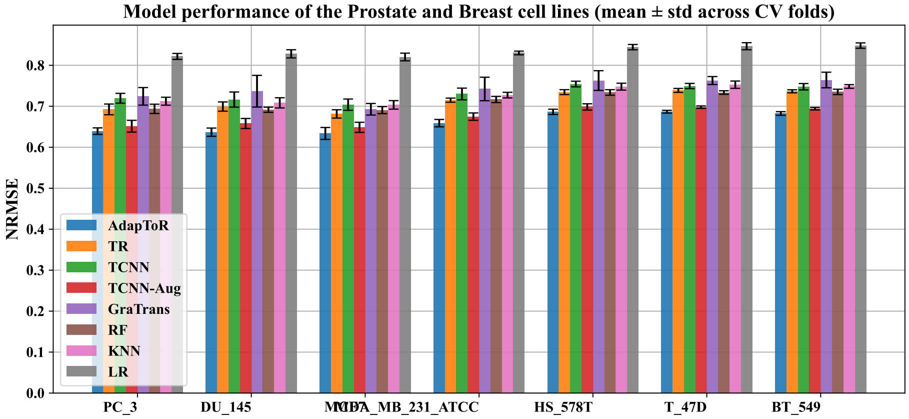

Figure S7: Bar plot showing cell line-wise model performance in terms of NRMSE for the cell lines corresponding to the prostate and breast cancer. Each bar represents the mean NRMSE across 5 cross-validation folds for a given cell line, and the error bars indicate the corresponding standard deviation.

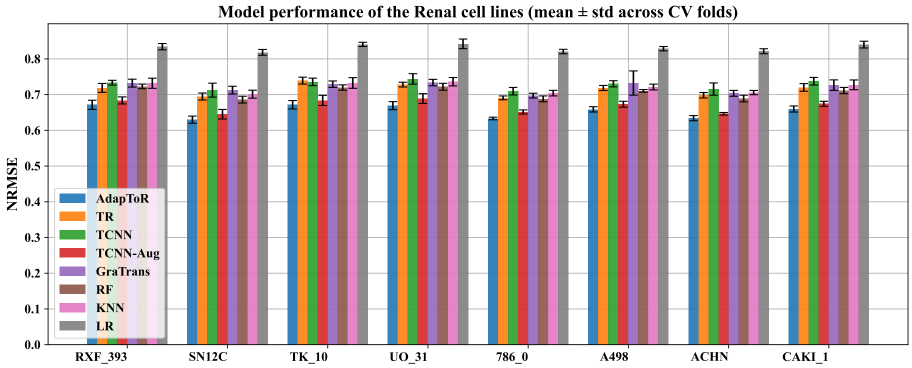

Figure S8: Bar plot showing cell line-wise model performance in terms of NRMSE for the cell lines corresponding to the renal cancer. Each bar represents the mean NRMSE across 5 cross-validation folds for a given cell line, and the error bars indicate the corresponding standard deviation.

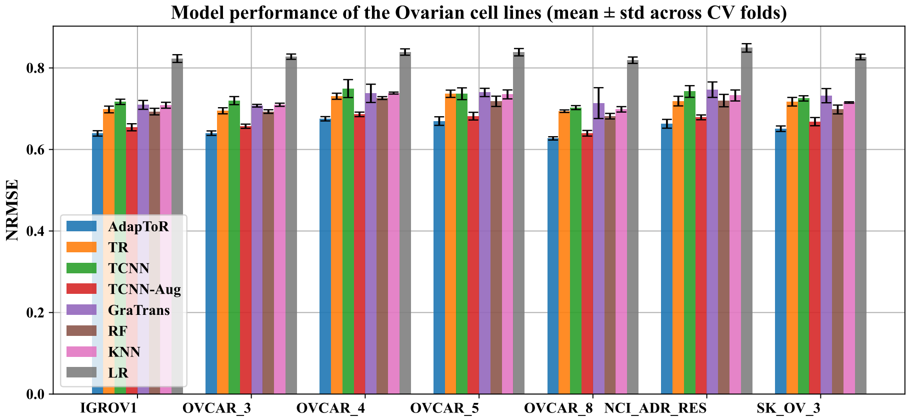

Figure S9: Bar plot showing cell line-wise model performance in terms of NRMSE for the cell lines corresponding to the ovarian cancer. Each bar represents the mean NRMSE across 5 cross-validation folds for a given cell line, and the error bars indicate the corresponding standard deviation.

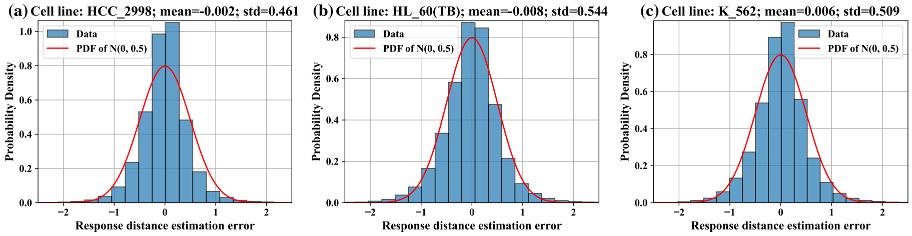

Figure S10: (a-c) Distributions of response distance estimation error of the test samples for three representative cell lines: (a) HCC2998, (b) HL-60 (TB), and (c) K562. As indicated in the titles, the mean and std values of the estimation error are around 0 and 0.5, respectively. Because the error distributions exhibit a bell-shaped pattern, we compared them with a Gaussian distribution (mean=0 and std=0.5), whose probability density function (PDF) is overlaid in each plot. It can be observed that the actual distributions are similar to the Gaussian distribution. To assess whether the magnitude of the estimation error correlates with actual response distances, Pearson's correlation coefficient (PCC) was computed. The PCC values are 0.086, 0.066, and 0.087 for cell lines HCC2998, HL-60 (TB), and K562, respectively.

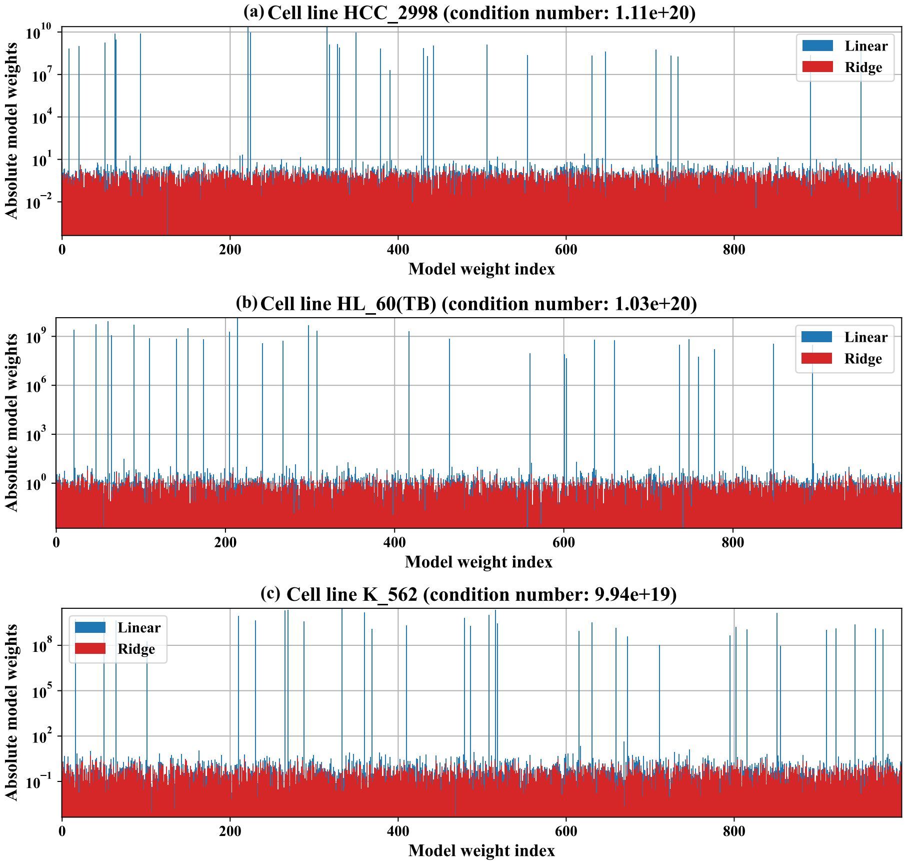

Figure S11: Absolute weights of linear TR models (blue) and Ridge TR models (red) trained on three representative cell lines: (a) HCC2998, (b) HL-60 (TB), and (c) K562. In each case, 60% of the training samples were selected as structure anchors, and the first 1,000 model weights were plotted. The figure titles indicate the condition number of the design matrix for each cell line. Notably, extraordinarily large condition numbers lead to unstable weights in the linear models, whereas the Ridge models have stable weights because of the L2 penalty.

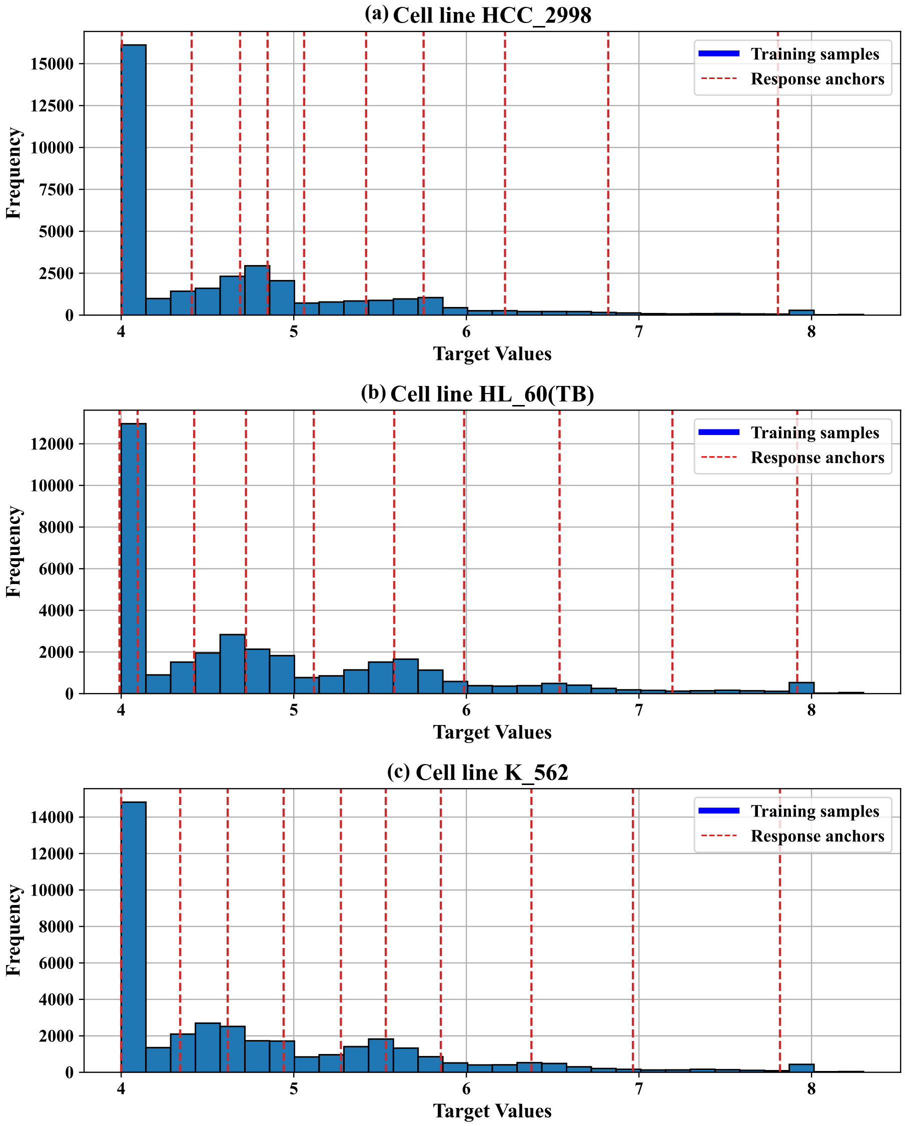

Figure S12: Histograms of response values for training samples for three representative cell lines: (a) HCC2998, (b) HL-60 (TB), and (c) K562. Dashed lines indicate response values of the 10 response anchors selected by K-means clustering.

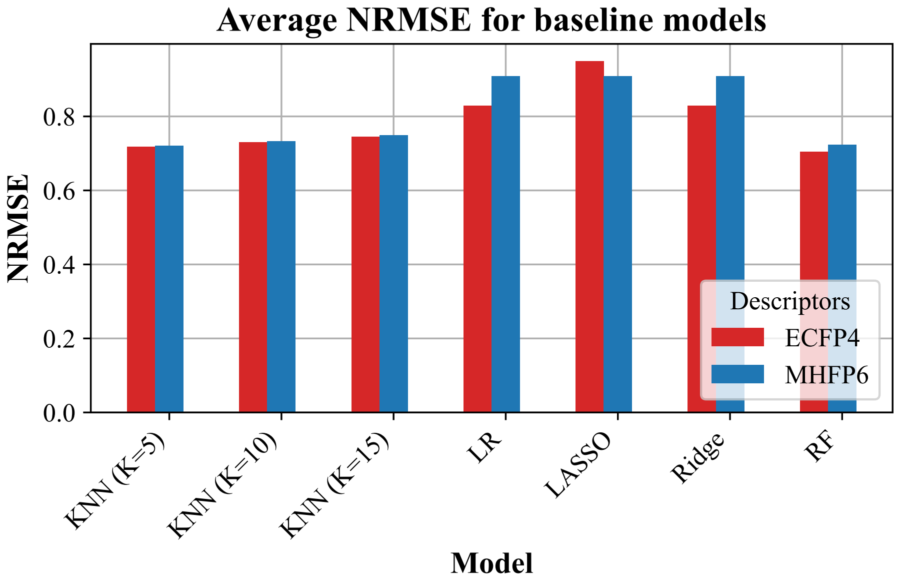

Figure S13: Average NRMSE of baseline models, including LR, LASSO, Ridge, RF, and KNN with ECFP4 and MHFP6 as input. The NRMSE values were averaged over 59 cell lines and 5 CV splits. For KNN, the number of nearest neighbors (K) was set to 5, 10, and 15. For LASSO and Ridge, the hyperparameter λ was set to 0.05. LR: Linear Regression, LASSO: Linear regression with L1-regularization, Ridge: Linear regression with L2-regularization, RF: Random Forest, KNN: k-nearest neighbor.

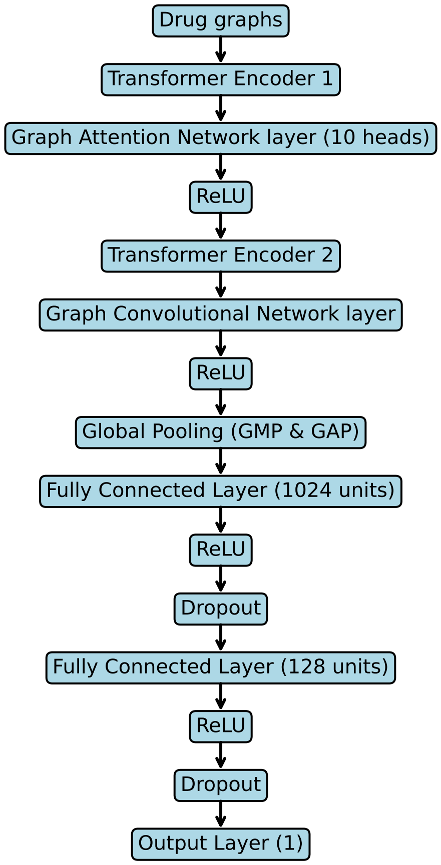

Figure S14: Model architecture of Graph Transformer adapted from [1].

References

[1] T. Chu, T. T. Nguyen, B. D. Hai, Q. H. Nguyen, and T. Nguyen. Graph Transformer for Drug Response Prediction. *IEEE/ACM Transactions on Computational Biology and Bioinformatics*, 20(2):1065–1072, 2023.

[2] R. Zhang, D. Nolte, C. Sanchez-Villalobos, S. Ghosh, and R. Pal. Topological regression as an interpretable and efficient tool for quantitative structure-activity relationship modeling. *Nature Communications*, 15(1):5072, 2024.
