# Supplementary figures and images for: AdapTor: Adaptive Topological Regression for quantitative structure–activity relationship modeling
Source: J Cheminform. 2025 Aug 28;17:128. doi: 10.1186/s13321-025-01071-8 (PMC12392520; doi:10.1186/s13321-025-01071-8)

**(a) NRMSE: AdapToR vs. TCNN-Aug**

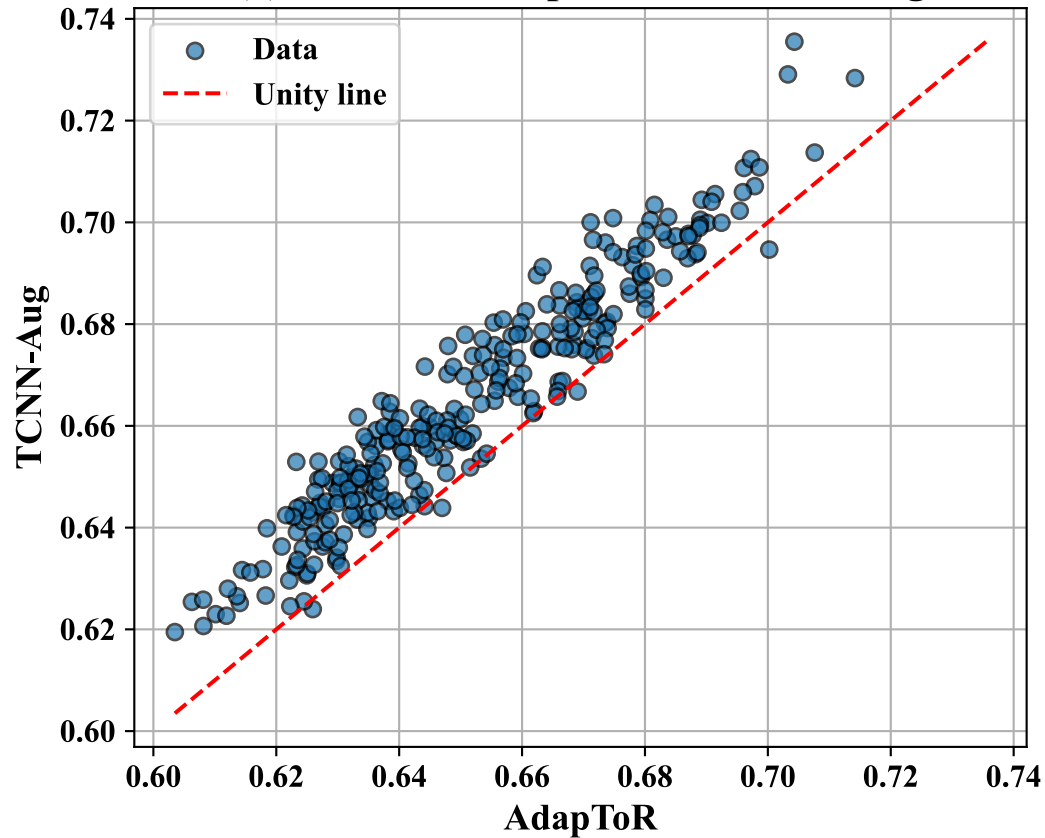

**(b) NRMSE: AdapToR vs. TR**

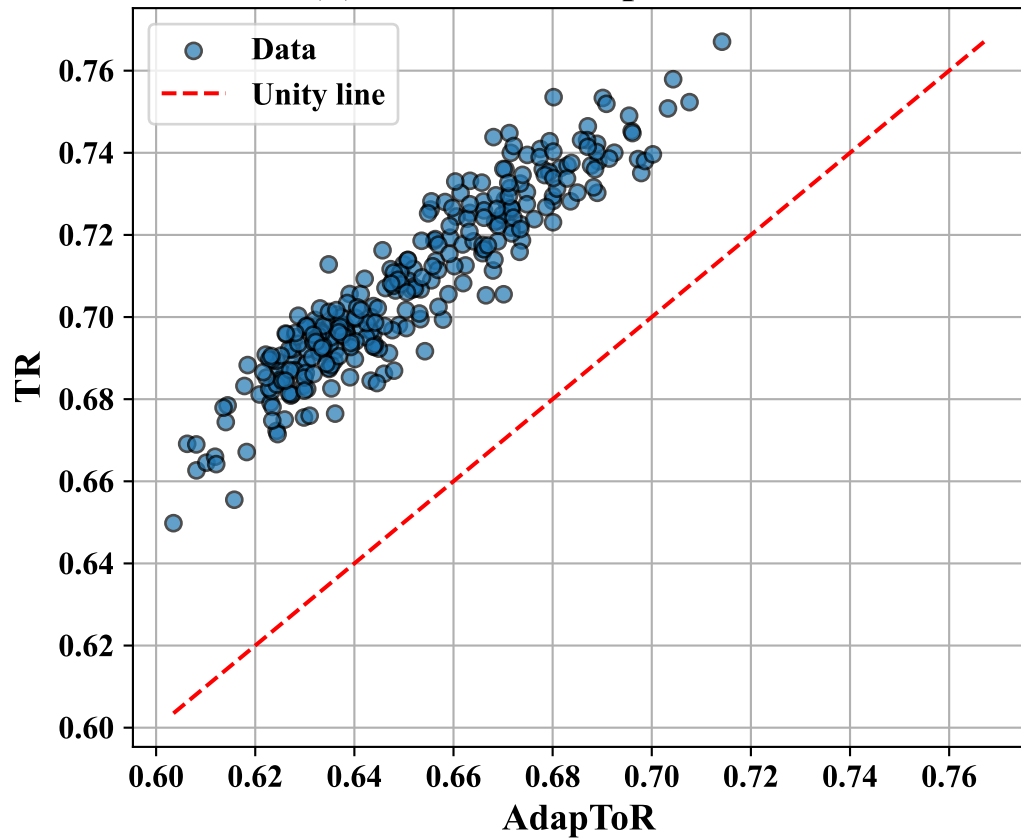

Supplement: Supplementary file 2 — Supplementary Material 2. [file 13321_2025_1071_MOESM2_ESM.pdf]
